# Mr. Stafford on Urethral Strictures

**Published:** 1829-07-01

**Authors:** 


### XXXIII.

### STRICTURES OF THE URETHRA.*

We have been often much surprised, that, in a disease of such frequent occurrence, and in one so dreadful in its effects as stricture, no improvements of any moment should have suggested themselves in its treatment besides that of the application of caustic.

At a time when the knife of the surgeon operates with confidence in situations and on organs for years deemed inaccessible, we were prepared to expect that a mechanical obstruction in a canal so easily examined as the urethra, should partake of the benefits of the advanced state of surgical science. We therefore took up Mr. Stafford's book with pleasing anticipations, and it is but justice to him to say, that we have not been disappointed.

The author first enters upon the consideration of strictures in general, and the only point we think it necessary to notice in this part of his work is the very proper importance he ascribes to the inflammatory foundation and tendency of almost every form of stricture.

Mr. S. then proceeds to the treatment of permanent stricture, and it is to his description of an instrument invented by him for dividing it that we would call the attention of our readers.

"The instrument for operating on permeable strictures (which for sake of distinction I have called the double lancetted stilette) consists of a round silver graduated sheath, open at both ends, of the size of No. 10 catheter, with rather a less curve, and of a stilette which is also hollow, and open at both ends. This stilette is furnished, at one end of it, with two oblong lancets, and at the other with a handle, resembling a button. When the instrument is complete, the stilette fits into the sheath, so that by pushing the handle the lancets will project from the extremity of the tube, and by drawing it back they will retire into it again. When used, (the mode of doing which will be presently explained,) the instrument is passed over a wire down to the stricture, and the lancets are thrust forward on each side of it, by which the contraction is made as large as the natural size of the urethra.* The armed stilette intended to divide impermeable strictures, exactly resembles the one just described, excepting that instead of the stilette being hollow it is solid, and in the place of two there is only one lancet.

Before using the instruments, the exact distance of the stricture from the extremity of the urethra should be ascertained. In the armed catheter, which is intended to divide strictures over the wire, which serves as a guide, the wire must be introduced through the stricture first. The mode of accomplishing this is by passing the smallest possible sized catheter made to contain the wire into the bladder. The wire which is double the length of the catheter, and blunted at one end, so that it may not injure the bladder, is then pushed forward, and the catheter gradually withdrawn, by which the former is left in the canal of the urethra. The armed catheter is then passed over the wire, until its point rests against the stricture, (which is known by means of the graduation,) and being held securely in such position, the handle of the stilette is pressed gently and gradually.

As soon as any impression is made, the lancets should be allowed to retire into their sheaths, and the blunt point of the instrument urged forward. If it do not pass on the lancets may be again used as before. After the stricture is divided, the armed catheter should be withdrawn, and its place supplied by one of elastic gum of the same size.

* A series of observations on strictures of the urethra, with an account of a new method of treatment, successfully adopted in cases of the most obstinate and aggravated form of that disease. Illustrated by Cases and a Plate. 2d Edition, by R. A. STAFFORD, M.R.C.S. &c. &c.

* This handle has hitherto been formed like a button; but I have thought it would be of advantage to have it made like two rings large enough to admit the finger and thumb, similar to the handle of a pair of scissors.

This should remain for a day or two, to prevent the reunion of the divided parts, and to preclude the possibility of extravasation of urine; and on its removal, a bougie should be passed twice in the week, or as often as may be judged necessary, for some time; and the same treatment adopted as for stricture in general. The armed stilette intended to divide impermeable strictures, must be used precisely in the same manner as the other, of course excepting the wire which cannot be introduced; and the same direction for the after treatment are necessary for both."

The objections to the use of these instruments, and more particularly of the single lancetted stilette, besides its novelty and boldness, is the apparent risk and uncertainty of its results. This, however, Mr. Stafford, in the preface to his second edition, answers most conclusively, and we cannot do better than quote his own words.

"With regard to the safety of the operation, a subject on which I am aware there exists much prejudice and doubt, even among some of the most eminent and liberal surgeons, I can only say, that I have operated more than twenty times, without the slightest dangerous symptom occurring at the time or afterwards;—that I have divided strictures in the urethra in almost every part of its course, at distances of one, three, four, five, and six inches from the orifice, at the point immediately behind the bulb, and throughout the whole membranous portion;—that some of these strictures have been half an inch, others an inch, and, in one case, two inches in length; that I operated at one time on four strictures in the same urethra, varying from one-fourth of an inch to above an inch in extent; and that in no instance was there a symptom to occasion the slightest alarm. The small quantities of blood lost from the operation was surprising, only in one case amounting to a table-spoonful, and usually not exceeding a few drops, or a tea-spoonful. This fact is so extraordinary, that unless there had been repeated proofs, it would hardly be credited. The inflammation which has occurred has never been very great; and when it has taken place, I am much inclined to attribute it to the irritation excited by the catheter having been left in the bladder. I am the more confirmed in this opinion from the fact, that in the only case in which I omitted its introduction no sensible inflammation followed.

"The superiority of the division by the lancetted stilettes over the only plan of treatment which can be brought in competition with it—that by the caustic—is evident from the following circumstances. The pain is much less. This was admitted by every patient who had experienced both plans of treatment. In truth, it is so little, as, by their own confession, to be not worth mentioning. As a proof of this, all my patients stood during the operation, which did not usually occupy a longer time than a period varying from one to two minutes. The bleeding is not so great as what often attends the passage of a common bougie, consequently very much less than that after the application of caustic, in which the loss of half a pint or a pint of blood is no uncommon occurrence. The formation of a false passage, which in the most experienced hands will inevitably sometimes occur, from the use of caustic bougies, has never resulted in any case where I have employed the instruments. The last, and perhaps principal proof of superiority, however, of this plan of treatment is, the shortness of time occupied, and the rapidity of the cure. The length of time necessary for the common method, of course varies indefinitely:—three months may be stated a short period, and it often extends to one or two years, with a great chance of the recurrence of the disease in a more aggravated form. On the contrary, the longest time which it has been found necessary to pass a bougie after dividing the stricture with the lancetted stilette, has never exceeded six weeks; and in those cases it was passed merely to satisfy myself and the patient of the non-existence of the disease. Usually a large sized bougie has been introduced immediately after the operation; and the cases have not required attendance more than three weeks or a month."

When we say that this statement is borne out by cases not selected, but, according to Mr. Stafford's assertion, simply laid before the medical public in the order in which they have occurred, we think that we should not have done our duty to our surgical readers if we had passed over his work without notice.

---

